# Occipital lobe epilepsy presenting as content‐specific reading‐induced seizures

**DOI:** 10.1002/epd2.70193

**Published:** 2026-03-26

**Authors:** Christopher M. Kyper, Lauren E. Chorny, William O. Tatum

**Affiliations:** ^1^ Department of Neurology Mayo Clinic Jacksonville Florida USA

**Keywords:** epilepsy, localization, reading, reflex, semiology, video‐EEG

## Abstract

Content available: Video.

Reading epilepsy is a rare form of reflex epilepsy that most often arises from the dominant temporoparietal cortices in reports, but likely involves the more widespread language network including visual, frontal, temporal, and parietal fields.^1^, ^2^ These seizures are reproducibly provoked by reading word text, though limited linguistic detail is available for provocative inventories involving a specific lexicon.^3^ Patients may manifest orofacial myoclonus, described as forced blinking, periocular twitching, aphasia, or stammering.^4^ Verbal expression, comprehension, and writing problems vary in severity though most patients with reading epilepsy have normal neurological examinations, imaging, and EEG. We report a unique case of content‐specific reading epilepsy originating in the occipital lobe.

A 19‐year‐old right‐handed female had rare convulsions and underwent video‐EEG for seizure classification. Over the past year, she experienced new‐onset of three bilateral tonic–clonic seizures, each triggered by reading without known seizure risk factors. Evaluation revealed a normal MRI brain, standard EEG, and neurological examination, and no epilepsy risk factors were identified. A voracious reader, she scaled back due to the anticipation of seizure precipitated by reading specific content, including a mathematics textbook and emotionally charged paperback novels containing complex historical and philosophical fictional narratives, with a known content trigger of *War and Peace* by Leo Tolstoy. Seizures consistently occurred within 10 min of reading onset. As a provocative measure to evaluate her stereotypical seizure, she was asked to silently read her new paperback copy of *War and Peace* by Leo Tolstoy. Within 5 min of reading onset, she reported her typical warning of a visual aura that evolved into clonic jerking of the right side of her mouth, tonic deviation of eyes and head to the right, repetitive forceful blinking, and right arm extension with left arm flexion, ultimately evolving into a bilateral tonic–clonic seizure and terminal unilateral left arm clonic jerking with left eye deviation. There was no evidence of oromandibular reflex myoclonus at seizure onset. She reported an aura of bilateral blurred vision followed by the “words floating off of the page to the right,” during which rare left posterior temporal slowing and left temporo‐occipital sharp waves were present. Ictal EEG revealed periodic left occipital spike‐and‐slow wave discharges evolving into 5–6 Hz left posterior quadrant rhythmic theta before rapid propagation to involve both hemispheres, resulting in crescendo‐decrescendo phasic myogenic artifact and left hemispheric post‐ictal slowing (Figure 1). She was started on antiseizure medications and has been seizure‐free since her Epilepsy Monitoring Unit admission (Video 1).

**FIGURE 1:**
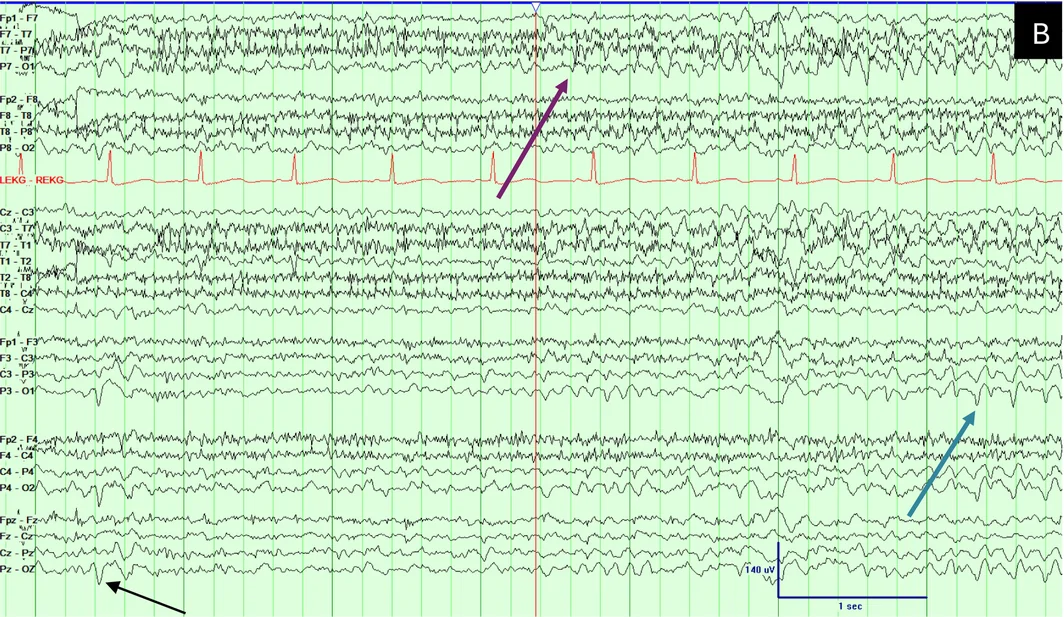
Electrographic focal seizure onset showing emergence of left hemispheric rhythmic ictal theta activity at P7‐O1 evolving to repetitive spiking over the left occipital region with a bilateral occipital predominant spatial field of distribution denoted by the blue arrows. Note the bi‐occipital sharp‐and‐slow wave in the first second (black arrow).

**VIDEO 1:** Video displaying seizure semiology of right head version progressing to generalized tonic‐clonic convulsions while reading *War and Peace* with associated ictal EEG with periodic left occipital spike‐and‐slow wave discharges evolving into 5–6 Hz left posterior quadrant rhythmic theta before rapid propagation to involve both hemispheres resulting in crescendo‐decrescendo phasic myogenic artifact and left hemispheric post‐ictal slowing. Video content can be viewed at https://onlinelibrary.wiley.com/doi/10.1002/epd2.70193.

This novel case highlights reading epilepsy originating in the occipital lobe, with seizures reproducibly triggered by semantically‐rich content requiring heightened visual and cognitive processing. Her seizures involved both complex visual and language processing in addition to visual‐concentration‐emotion axes with a latency involving the dominant occipital language network. Most documented cases of reading‐induced focal seizures are confined to the dominant temporal or temporoparietal regions, although seizures may also occur with alternative onset regions within the reading network or cause generalized seizures.^2^ Our patient experienced a positive visual aura, differing from the typical negative visual or language phenomena. Visual auras are usually described as bright or black spots in the visual fields, or words and letters switching places or moving from their site of origin and language impairment.^5^ Our case supports a continuum for reading epilepsies of occipital onset confirmed by video‐EEG monitoring to extend the spectrum of focal reading epilepsies to emphasize the impact of semantic activation of visual–spatial connections. It has been theorized that reading epilepsy occurs from networks integrating visual systems and language processing. We speculate that, unlike other cases of reading epilepsy, our patient had visual input as the primary generator triggering the same intrinsic network arising within the posterior quadrant of the brain. Complex visual processing is thought to be derived from the ventral visual stream, which could explain a propagation path within this occipitotemporal network.^1^, ^6^ Although described with facial expression recognition, a short‐term synaptic strengthening via back‐projections from the posterior temporal lobe to the occipital lobe could explain a pathway of strengthening visual awareness and perception and thus a path for occipital‐onset reading epilepsy.^7^ Similar to triggering visual evoked potentials using a checkerboard pattern shift reversal, our patient's seizures were always induced by saccadic reading movements during specific content and formatting (small font size, high‐contrast black‐and‐white paperback) necessitating complex visual, spatial, and language‐associated cognitive processing.^8^ Previous reports include specific languages that correlated the complexity with seizure provocation.^2^ In summary, we describe a novel case of reading epilepsy of occipital origin, triggered by a combination of visual formatting and semantic complexity confirmed by video‐EEG, implicating a rare localization, underscoring a spectrum of networks involving reflex epilepsies triggered by reading.

## AUTHOR CONTRIBUTIONS

Drafting of the manuscript, acquisition of data, manuscript preparation, and literature review. Acquisition of data, manuscript preparation, literature review, and critical revision of the manuscript. Study concept and design, acquisition of data, critical revision of the manuscript for important intellectual content.

## FUNDING INFORMATION

No funding was secured for this study.

## CONFLICT OF INTEREST STATEMENT

The authors have none to declare. This work has not previously been published or presented.

**Test yourself**

1. What location do reading epilepsies commonly arise?
  - Frontal lobe
  - Dominant temporoparietal
  - Occipital lobe
  - Nondominant parietal lobe
  - Nondominant temporal lobe
2. What auras are commonly associated with reading epilepsies?
  - Negative visual aura of bright/black spots in visual field
  - Positive visual aura of words floating from the page
  - Déjà vu
  - Epigastric rising
  - Auditory hallucinations
3. The proposed pathophysiologic mechanism for seizure generation in an occipital lobe‐onset reading epilepsy most likely involves which neural network?
  - Dorsal visual stream with parietal‐frontal projections
  - Ventral visual stream with occipitotemporal language integration
  - Limbic network involving hippocampal‐amygdala circuits
  - Primary motor cortex with supplementary motor area activation.

*Answers may be found in the* *Supporting Information*.

## Supporting information


Appendix S1.

Appendix S1.

## Data Availability

Data sharing not applicable to this article as no datasets were generated or analyzed during the current study.
