# Supplementary material for: Occipital lobe epilepsy presenting as content‐specific reading‐induced seizures
Source: Epileptic Disord. 2026 Mar 26;28(4):1294–7. doi: 10.1002/epd2.70193 (PMC13499256; doi:10.1002/epd2.70193)
Supplement: Supplementary file 1 [file EPD2-28-1294-s001.docx]

Test yourself Answers:

1.b

2.a

3.b
